# Morphological and Spectroscopic Characterization of Multifunctional Self-Healing Systems

**DOI:** 10.3390/polym17101294

**Published:** 2025-05-08

**Authors:** Liberata Guadagno, Elisa Calabrese, Raffaele Longo, Francesca Aliberti, Luigi Vertuccio, Michelina Catauro, Marialuigia Raimondo

**Affiliations:** 1 Department of Industrial Engineering, University of Salerno, Via Giovanni Paolo II, 132, 84084 Fisciano, Italy; lguadagno@unisa.it (L.G.); elicalabrese@unisa.it (E.C.); rlongo@unisa.it (R.L.); faliberti@unisa.it (F.A.); 2 Department of Engineering, University of Campania “Luigi Vanvitelli”, Via Roma 29, 81031 Aversa, Italy; luigi.vertuccio@unicampania.it (L.V.); michelina.catauro@unicampania.it (M.C.)

**Keywords:** tunneling atomic force microscopy (TUNA), epoxy resins, multiwall carbon nanotubes (MWCNTs), molecular self-healing fillers, multifunctional nanocomposites, reversible interactions

## Abstract

Multifunctional self-healing supramolecular structural toughened resins, formulated to counteract the insulating properties of epoxy polymers and integrating auto-repair mechanisms, are morphologically and spectroscopically characterized using Tunneling Atomic Force Microscopy (TUNA) and Fourier transform infrared spectroscopy (FT-IR), respectively. Specifically, the multifunctional resin comprises self-healing molecular fillers and electrically conductive carbon nanotubes (CNTs) embedded in the matrix. The selected self-healing molecules can form non-covalent bonds with the hydroxyl (OH) and carbonyl (C=O) groups of the toughened epoxy matrix through their H-bonding donor and acceptor sites. An FT-IR analysis has been conducted to evaluate the interactions that the barbiturate acid derivatives, serving as self-healing fillers, can form with the constituent parts of the toughened epoxy blend. Tunneling Atomic Force Microscopy (TUNA) highlights the morphological characteristics of CNTs, their dispersion within the polymeric matrix, and their affinity for the globular rubber domains. The TUNA technique maps the samples’ electrical conductivity at micro- and nanoscale spatial domains. Detecting electrical currents reveals supramolecular networks, determined by hydrogen bonds, within the samples, showcasing the morphological features of the sample containing an embedded conductive nanofiller in the hosting matrix.

## 1. Introduction

The morphological and spectroscopic evaluations of multifunctional epoxy composites, performed using TUNA and FT-IR, are the primary focus of this research. Multifunctional self-healing epoxy resins can be formulated to counteract the insulation properties of epoxy polymers by incorporating self-healing fillers together with electrically conductive nanofillers. It is well known that the presence of conductive fillers in the polymeric matrices can confer many functional properties to the final material [1,2,3,4,5,6,7]. Self-healing functional resins have recently been proposed in the literature and represent alternative materials to thermosetting microencapsulated systems suitable for use as structural materials [8,9,10,11,12,13,14,15,16].

The design of this kind of self-healing structural polymer heavily relies on concepts from chain dynamics and polymer physics [17,18,19]. In the previous papers, CNTs were functionalized with thymine-based ligands (CNT-t) and barbituric acid (CNT-b) using a copper(I)-catalyzed alkyne/azide cycloaddition (CuAAC) click reaction to design materials with dynamic characteristics. Functionalized carbon nanotubes (CNT-t and CNT-b) were dispersed into an aerospace-grade epoxy formulation toughened by the reaction between the carboxyl groups of a carboxyl-terminated butadiene–acrylonitrile liquid rubber (CTNB) and the epoxy groups of tetraglycidylmethylenedianiline (TGMDA). Due to the strong and attractive interactions between the rubber phase and the CNT walls, the functionalization process enables CNT bridges to pass through the epoxy matrix. A self-repair efficiency of 50% was observed for both functional groups, namely barbiturate- and thymine-based moieties [8,9]. Generally, to design self-healing materials that maintain the mechanical properties of high-performance structures, chemicals typical of load-bearing structures were proposed in the literature [8,9,13,20]. It has been demonstrated that the electrical percolation threshold (EPT) of self-healing resins containing functionalized CNTs increases with the functionalization of carbon nanotubes [8,21,22].

This behavior is most likely due to the different morphological parameters of the functionalized carbon nanotubes and the different CNT distribution determined by the functionalization. In refs. [8,9], functional groups covalently linked to the carbon nanotubes activate self-healing mechanisms. Alternatively, fillers/molecules with a chemical structure that allows them to anchor themselves in a suitable resin and create reversible hydrogen bridges can be used to impart the self-healing function to the resin [23]. These self-healing fillers are molecules or compounds containing structural groups capable of donating and accepting hydrogen bonds. Furthermore, they are compatible with the hosting polymer matrices and can form strong, reversible, and attractive interactions. They readily produce the cumulative effects of reversible interactions based on hydrogen bonding in the epoxy matrix [24,25,26]. Carbon nanotubes (CNTs) or other structured forms of carbon can be incorporated into the polymer matrix to impart electrical conductivity to the material [27,28,29,30]. The presence of the nanofiller may also be beneficial for forming interphase regions between the filler and the polymeric matrix (for instance, the epoxy matrix in contact with the CNT or graphene layer walls) [31,32,33,34]. The existence of this interphase enables various materials to work in concert with self-healing fillers while also reducing the occurrence of microcracks. Using unfunctionalized carbon nanotubes (CNTs) combined with self-healing fillers represents a valid alternative for developing multifunctional resins. This approach also allows for a good distribution of carbon nanotubes due to the interactions of the self-healing filler with the polar group of the resins and the defects on carbon fillers [23]. Furthermore, this approach preserves the structural quality of CNTs that is related to the aspect ratio or the length-to-diameter ratio [8,35]. The CNT length can be shortened due to functionalization [9,36,37]. This can be undesirable for applications that need larger aspect ratios, such as in producing polymeric composites [38,39]. The significant drop in electrical conductivity observed in the self-repairing samples containing modified carbon nanotubes may be partially attributed to this phenomenon [40]. The goal of this work is to assess the significant innovation represented by the use of unfunctionalized carbon nanotubes (CNTs) in combination with self-healing fillers to formulate toughened self-healing supramolecular resins based on hydrogen bonding, as determined through morphological and spectroscopic analyses. By measuring the fA/pA tip/sample currents and identifying the distribution of the conductive filler, TUNA enables the morphological characterization of the conductive samples. The TUNA images’ lateral resolution is almost equivalent to the tip radius’s end (20 nm). The clearest areas in the TUNA photos show the more conductive zones. Before undergoing examination via TUNA, the nanocomposites described in this paper were subjected to etching to highlight the morphological features of unfunctionalized carbon nanotubes, as well as their distribution within the polymer matrix and their interaction with the globular rubber domains. It is noteworthy that the TUNA technique offers valuable supplementary insights into the conductivity of nanoscale domains, as it is capable of detecting currents while also providing information regarding the intricate morphological characteristics of the conductive filler embedded within the host matrix [41,42,43]. Unfunctionalized carbon nanotubes, in combination with self-healing fillers, show a good dispersion in the resin.

The performed characterizations evidence that this specific combination can provide good solutions for achieving “smart” and “multifunctional” resins with a high Tg, utilizing commercially available materials without the need for synthesis procedures, thereby reducing the time and costs associated with the composite formulation.

## 2. Materials and Methods

### 2.1. Materials

The selected self-healing fillers are structurally characterized by suitable functional groups that can form reversible non-covalent bonds with the polymer matrix and each other. The formulated smart materials are composed of an epoxy matrix loaded with unfunctionalized multiwall carbon nanotubes (CNTs) and self-healing fillers. The information about the single components used to formulate the samples investigated is illustrated in Table 1.

By forming a hydrogen bond with the epoxy matrix they interact with, the self-healing molecules can more effectively activate the auto-repair mechanism that relies on reversible non-covalent bonds [9,45,46,47]. Before the curing stage, they had been dissolved/distributed throughout the epoxy matrix.

**Figure 1:**
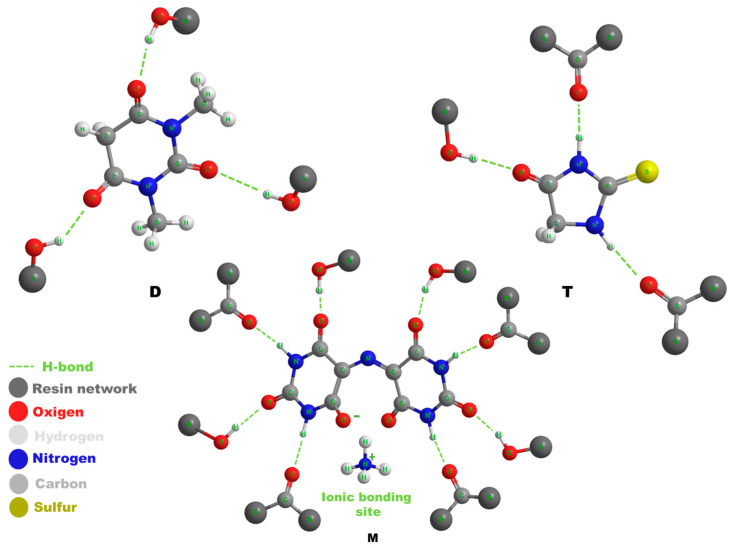
A schematic model showing the chemical structures of the healing molecules D, T, M and the hydrogen bonding interactions that they establish with the carbonyl groups (H-bond acceptor sites) and the hydroxyl groups (H-bond donor sites) of the toughened matrix.

Figure 1 shows a schematic model of the chemical structures of the healing molecules and the hydrogen bonding interactions they establish with the carbonyl groups (H-bond acceptor sites) and the hydroxyl groups (H-bond donor sites) of the toughened matrix. The scheme shows the reversible attractions based on hydrogen bonds with dashed segments.

Table 2 presents the composition and description of the characteristics of the analyzed samples, along with the associated acronyms.

The preparation of all samples involved using an E/B ratio equal to 80/20. In the samples containing the elastomeric phase C, the latter was added at a concentration of 12.5 phr with respect to E, and it was covalently bonded to the epoxy precursor through a reaction catalyzed by triphenylphosphine (PPh3), used at a concentration of 10 phr with respect to E. The hardening agent H was solubilized in an amount of 55 phr with respect to E, while the carbon nanotubes and the self-healing fillers were added, respectively, in the weight percentage of 0.5 wt% and 1.0 wt% with respect to the Ep epoxy sample, whose composition is E + B + C + H.

The experimental procedures for formulating the samples and covalently functionalizing the epoxy precursor through the rubbery phase have already been reported in the literature [9,23,45]. It was demonstrated that by reducing the stiffness of the tetrafunctional epoxy precursor and facilitating the arrangement of hydrogen bonding interactions through the activation of an auto-repair function, the presence of a rubber phase enables the formulation to host self-healing mechanisms.

The very low addition of carbon nanofillers makes the samples electrically conductive, allowing them to reach the electrical percolation threshold (EPT) [45].

### 2.2. Methods

#### 2.2.1. FT-IR

A Bruker Vertex 70 FT-IR spectrophotometer (Bruker Optics Inc., Billerica, MA, USA) was used to perform absorbance FT-IR measurements in the 4000–400 cm^−1^ range with a resolution of 2 cm^−1^, using 32 scans. More information is contained in ref. [8].

#### 2.2.2. FESEM

The formulated samples were morphologically characterized by FESEM (mod. LEO 1525, Carl Zeiss SMT AG, Oberkochen, Germany). A sledge microtome was used to cut sample sections from solid samples. These slices were etched before the FESEM observation [9].

#### 2.2.3. TUNA

Data regarding the multifunctional nanocomposites’ topography and local electrical current were acquired using the TUNA method.

To obtain repeatable results, several regions of the specimens were scanned. The images were analyzed using the Bruker software Nanoscope Analysis 1.80 (Build R1.126200).

Ref. [48] provides details on the acquisition parameters. The sample slices were etched before the TUNA observation [9].

#### 2.2.4. Self-Repairing Efficiency Evaluation Tests

The self-healing performance (*SH*) of all epoxy-cured samples was evaluated using Tapered Double Cantilever Beam (TDCB) fracture tests, following a method presented in a previous publication by the authors [45]. These tests enabled the determination of the critical fracture load values for both the unaltered samples ($P_{0}$) and the repaired samples after 24 h ($P_{H}$), and the ratio of these two parameters was used to compute the healing efficiency of the materials, as described in Equation (1):

(1) $$SH=\frac{P_{H}}{P_{0}}\times 100$$

## 3. Results and Discussion

### 3.1. FT-IR Analysis

An FT-IR analysis has been conducted to evaluate the interactions that barbiturate acid derivatives may form with the components of the toughened epoxy blend. One effective method for identifying functional groups in compounds is Fourier transform infrared (FT-IR) spectroscopy [49]. The molecular association is one of the variables that might cause a functional group’s absorption frequency to vary from its theoretical value [50]. The molecule segments within a sample can create bonds with other components by introducing functional groups. Specifically, they can create intramolecular and intermolecular hydrogen bonds.

The molecules under investigation can facilitate hydrogen bonding with the reinforced matrix due to their donor and acceptor sites, akin to those in the matrix (carbonyl and hydroxyl groups).

The FT-IR investigation was conducted to evaluate how hydrogen bond interactions affect the bands belonging to the characteristic functional groups of self-healing molecules (mainly carbonyl C=O groups) and the stretching vibration signal of the hydroxyl group present in the cured resin.

Table 3 presents the chemical formulas of the self-healing fillers to help clarify the FT-IR spectra.

#### 3.1.1. FT-IR Investigation of System Based on Filler D

**Figure 2:**
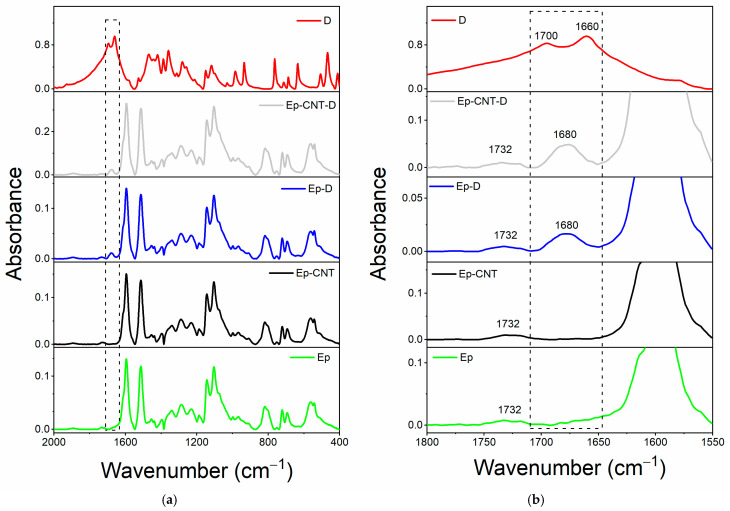
FT-IR spectra of the samples: Ep (green curve), Ep-CNT (black curve), Ep-D (blue curve), and Ep-CNT-D (light gray curve); filler D (red curve): (**a**) in the wavenumber range of 2000–400 cm^−1^ and (**b**) in the enlarged wavenumber range of 1800–1550 cm^−1^.

Figure 2a shows the comparison between the spectrum of filler D alone and the spectra of the oven-cured epoxy formulations, both loaded and unloaded with filler D, in the range of wavenumbers between 2000 and 400 cm^−1^. We can more clearly see that only the spectra of the samples containing the filler D have a distinctive peak around 1680 cm^−1^, thanks to the magnification in Figure 2b’s wavenumber range of 1800–1550 cm^−1^. The imide carbonyl C=O stretching band of the barbiturate acid derivative D may cause this signal, which falls within the carbonyl group’s frequency range (see chemical structure in Table 3). Two peaks associated with the carbonyl groups C=O may be seen in the area between 1700 and 1650 cm^−1^ in the spectra of filler D alone (see red curve). Due to the differing chemical environment, the identical functional group exhibits two distinct bands: an imide C_1_=O stretching group at approximately 1700 cm^−1^ and an imide C_2_=O stretching group at around 1660 cm^−1^ [51]. For cyclic imides with six carbon atoms, the typical value of the C=O stretching bond is around 1710–1700 cm^−1^; however, the presence of two nitrogen atoms bound to the C_2_=O group shifts the signal to lower frequencies for inductive effects [52,53]. These two signals converge into one broad peak around 1680 cm^−1^ when the filler is added to the toughened epoxy formulations Ep-D and Ep-CNT-D.

**Figure 3:**
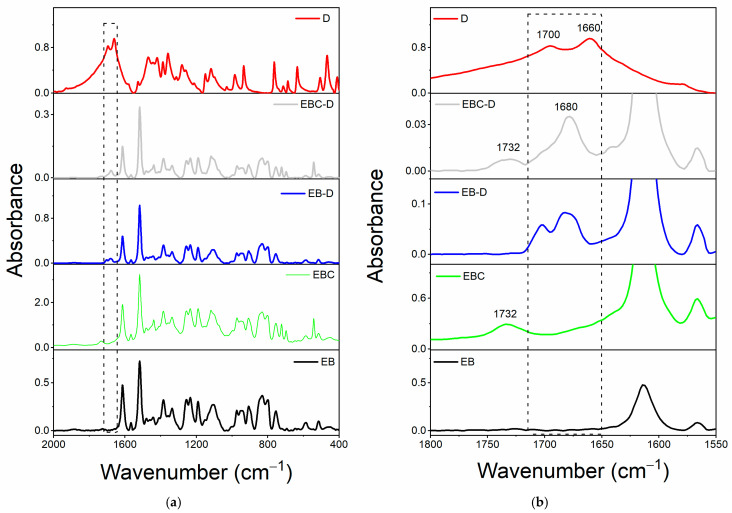
FT-IR spectra of the uncured liquid formulations: EB (black curve), EBC (green curve), EB-D (blue curve), and EBC-D (light gray curve); filler D (red curve): (**a**) in the wavenumber range of 2000–400 cm^−1^ and (**b**) in the enlarged wavenumber range of 1800–1550 cm^−1^.

To perform a more detailed evaluation, a spectral investigation of the uncured liquid formulations, both loaded and unloaded with filler D, has been conducted (Figure 3a). The wavenumber range of 1800–1550 cm^−1^ is enlarged in Figure 3b. It illustrates that adding filler D to the EB liquid mixture maintains the separation of the two peaks. Conversely, when filler D is incorporated into the toughened epoxy liquid matrix EBC, the peak near 1700 cm^−1^ shifts to a lower frequency, resulting in a single, broader signal.

In light of the above, it can be hypothesized that this change in the band shape of the C=O group is a clear demonstration of the involvement of this functional group in hydrogen bonding interactions (as an acceptor) with the O-H groups of the toughened EBC mixture. It is worth noting that the toughening reaction between the epoxy precursor E and the rubber phase C results in the generation of ester carbonyl groups, whose signal appears at 1732 cm^−1^ (see Figure 3b), and OH groups, which further increase with the resin curing process [9,23,45].

The EB liquid mixture does not contain hydroxyl groups, and for this reason, the two carbonyl signals of filler D are most likely still separated, as they cannot be involved in H-bond interactions.

Finally, it is worth noting that the signal at 1732 cm^−1^ can also be observed in the spectra of the Ep-based cured samples, as illustrated in Figure 2b.

#### 3.1.2. FT-IR Investigation of System Based on Filler T

**Figure 4:**
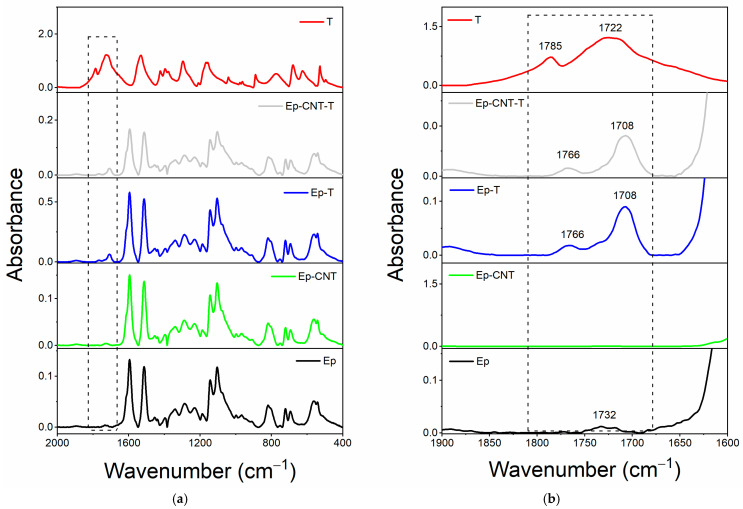
FT-IR spectra of the samples: Ep (black curve), Ep-CNT (green curve), Ep-T (blue curve), and Ep-CNT-T (light gray curve); filler T (red curve): (**a**) in the wavenumber range of 2000–400 cm^−1^ and (**b**) in the enlarged wavenumber range of 1900–1600 cm^−1^.

Figure 4a compares the spectrum for the single T filler alongside the spectra of the oven-cured epoxy formulations, both with and without the T filler, within the wavenumber range of 2000 to 400 cm^−1^. The enlarged view of the wavenumber range from 1900 to 1600 cm^−1^, illustrated in Figure 4b, reveals that only the spectra of the samples containing the T filler (Ep-T and Ep-CNT-T) exhibit two distinct peaks at approximately 1766 and 1708 cm^−1^. Concerning the FT-IR spectrum of the T filler alone (refer to the red curve in Figure 4), two peaks are observed at around 1785 and 1722 cm^−1^, which are attributed to the C=O stretching band of the carbonyl functional group present in T.

The filler T may be classified as a cyclic amide with five atoms (a γ-lactam). For this category of compounds, the FT-IR signal corresponding to the C=O bond typically appears in the range of 1750–1700 cm^−1^ (see Table 3 for chemical structure) [54]. There are two signals for the same carbonyl group because the T molecule has both H-bond donor (N-H) and acceptor (C=O) groups, which can interact with each other. Thus, the band at lower wavenumber values is due to C=O groups involved in intermolecular H-bond interactions with the N-H groups of another 2-thiohydantoin molecule, while the signal at higher wavenumbers could be due to free carbonyl groups [55,56,57,58]. When the filler is present in the cured epoxy formulation, these two peaks are shifted to lower wavenumbers, most likely due to the establishment of new H-bond interactions with the host epoxy matrix. In particular, some C=O groups could be involved in H-bonds with N-H groups of other T molecules, while other carbonyls could interact via H-bonding with the hydroxyl groups of the thermoset matrix.

**Figure 5:**
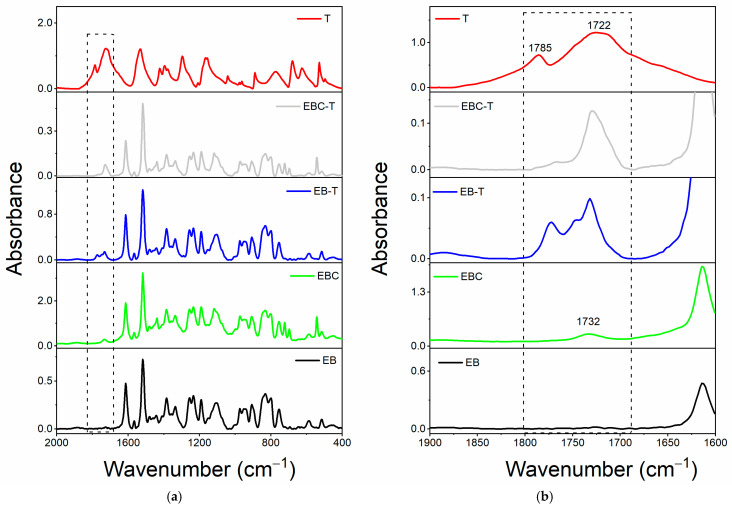
FT-IR spectra of the uncured liquid formulations: EB (black curve), EBC (green curve), EB-T (blue curve), and EBC-T (light gray curve); filler T (red curve): (**a**) in the wavenumber range of 2000–400 cm^−1^ and (**b**) in the enlarged wavenumber range of 1900–1600 cm^−1^.

Concerning the evaluation carried out on the liquid epoxy mixtures (Figure 5), it can be observed that only when the filler T is added to the toughened liquid blend EBC, the carbonyl group shows a single broad band (see light gray curve of EBC-T sample), while when it is dispersed within the EB blend, two signals are still detectable (see blue curve of the EB-T sample), as the EB blend does not show H-bond donor groups.

Similarly, for this system, it can be assumed that the self-healing filler T interacts with the toughened epoxy matrix through hydrogen bonding.

#### 3.1.3. FT-IR Investigation of System Based on Filler M

In the comparison made for filler M, it was not possible to determine if the same trend is observed. The eventual presence of any signal shift is likely not discernible because the bands of filler M overlap with those of the hosting matrix.

**Figure 6:**
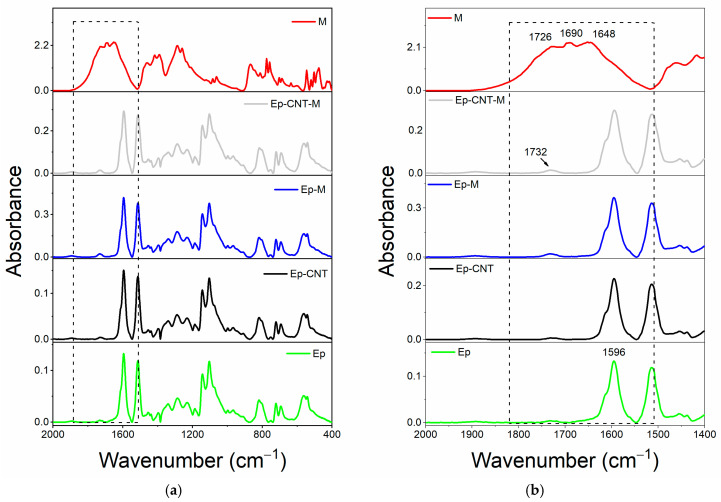
FT-IR spectra of the samples: Ep (green curve), Ep-CNT (black curve), Ep-M (blue curve), and Ep-CNT-M (light gray curve); filler M (red curve): (**a**) in the wavenumber range of 2000–400 cm^−1^ and (**b**) in the enlarged wavenumber range of 2000–1400 cm^−1^.

**Figure 7:**
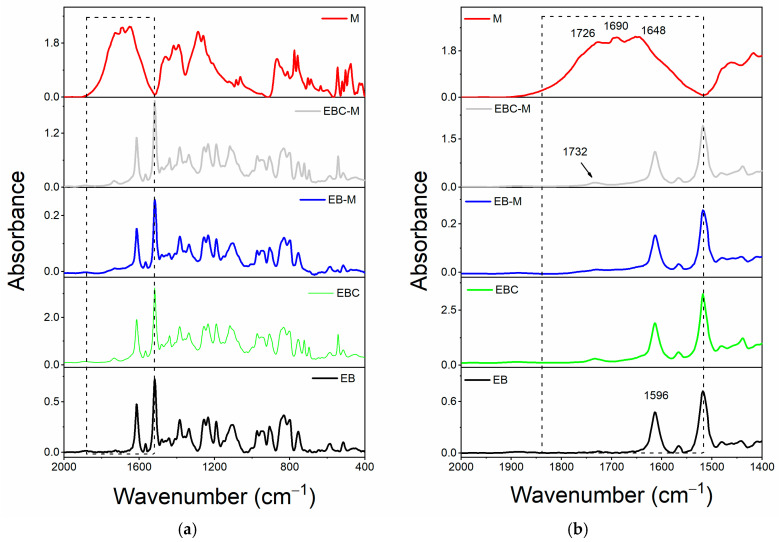
FT-IR spectra of the uncured liquid formulations: EB (black curve), EBC (green curve), EB-M (blue curve), and EBC-M (light gray curve); filler M (red curve): (**a**) in the wavenumber range of 2000–400 cm^−1^ and (**b**) in the enlarged wavenumber range of 2000–1400 cm^−1^.

Figure 6 and Figure 7 show that the characteristic absorbance bands of the three different carbonyl groups of the filler M (at 1726, 1690 and 1648 cm^−1^, see red curve) are not observable in the spectra of both cured epoxy samples Ep-M and Ep-CNT–M (see Figure 6a,b) and liquid epoxy mixtures EB-M and EBC-M (see Figure 7a,b), since these signals fall in the same wavenumber region of the ester carbonyl at 1732 cm^−1^ and other characteristic signals belonging to the epoxy precursor, such as the band at 1596 cm^−1^ attributed to the stretching vibration of the benzene ring [59,60,61].

### 3.2. Additional FT-IR Analysis

Additional FT-IR analyses were conducted on the cured samples to better illustrate the development of H-bonding interactions between the epoxy matrix and the self-healing fillers. Specifically, a thorough analysis of the absorbance bands of the OH groups has been conducted for the systems that contain both the CNT and self-healing fillers. Due to the intermolecular hydrogen bonds formed during the curing cycle, the O-H stretching signal (3200–3650 cm^−1^) of an epoxy resin in the solid state appears as a broad band [62]. Nonetheless, information regarding the type of hydrogen bonding interactions can be gleaned from the band profile in the vicinity of the hydroxyl groups, as well as the presence of more or less pronounced shoulders and/or additional peaks [63].

**Figure 8:**
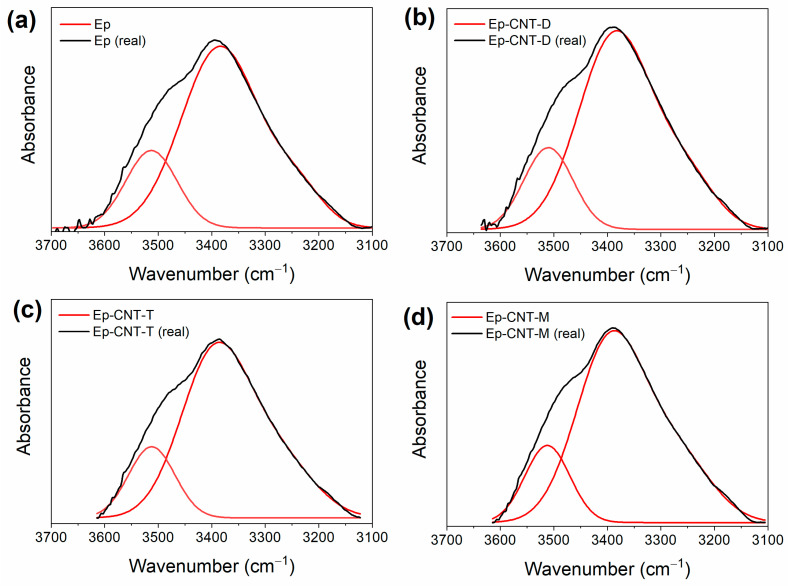
The deconvolution performed on the O-H absorbance band of the FT-IR spectra of the samples: (**a**) Ep; (**b**) Ep-CNT-D; (**c**) Ep-CNT-T; and (**d**) Ep-CNT-M.

A deconvolution has been applied to the signal in the frequency range of 3600–3100 cm^−1^ to examine the O-H stretching band profile in more detail. A mixed Gauss–Lorentz line shape has been utilized as the peak function, determined by fitting the data using a non-linear curve that considers the height, full width at half maximum (FWHM), and location of each component separately. This assessment technique has also been employed in the literature [64,65]. For each O-H stretching band (refer to the black curve, representing the actual peak, in Figure 8), this process resulted in the identification of two distinct peaks (illustrated by the red curves in Figure 8): one peak at elevated frequencies, associated with free hydroxyl groups, and another peak, which appears at lower frequencies, corresponding to O-H groups participating in hydrogen bonding interactions as donors. A quantitative evaluation of the hydrogen bonding interactions present in the composites was facilitated by the ratio R, defined as the area of the bonded O-H signal (A_OH-bond_) divided by the area of the free O-H signal (A_OH-free_). An increased value of this ratio indicates a higher number of OH groups engaged in hydrogen bonding interactions with self-healing fillers.

**Figure 9:**
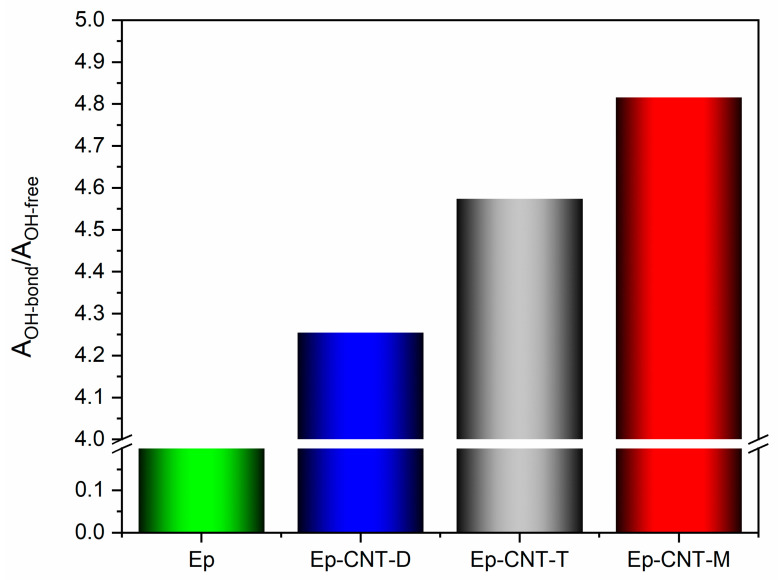
Values of the ratio R = A_OH-bond_/A_OH-free_ for the different formulations.

While Figure 9 displays the values of the ratio R = A_OH-bond_/A_OH-free_ for the various formulations, Figure 8 shows the outcomes of the deconvolutions. In particular, the composite loaded with 1.0 weight percent of the filler M has more H-bonding interactions due to the presence of the self-healing fillers, which favors the creation of a supramolecular network that can encourage the activation of self-healing mechanisms [45].

### 3.3. Morphological Investigation by FESEM and TUNA

**Figure 10:**
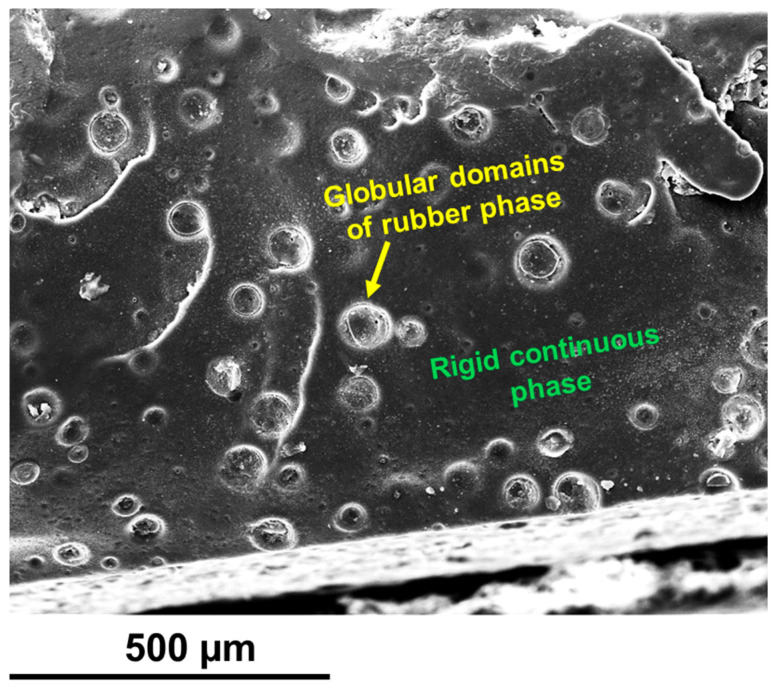
An FESEM image of the etched slice of the unfilled epoxy sample Ep.

FESEM and TUNA analyses were conducted on etched epoxy samples to assess the distribution of carbon nanotubes and self-healing fillers within the toughened polymer matrix modified with rubber domains. Additionally, reversible self-healing interactions were investigated, as the molecules’ structures contained groups that could facilitate the formation of hydrogen bonds between themselves and the epoxy matrix. A closer view of the fillers’ dispersion state is provided by the etching process, which partially removes the surface layers of the epoxy matrix. Furthermore, to prevent any movement of the rubbery phase chains, the surface of the examined samples was achieved through fracturing in liquid nitrogen [9]. The FESEM picture of the toughened epoxy sample, Ep, devoid of CNTs and self-healing fillers is displayed in Figure 10. On the etched slice surface of the unfilled epoxy sample Ep, we can clearly see the presence of globular domains of the rubber phase, highlighting their good distribution and uniform diameters within the rigid continuous phase.

The identification of globular domains was facilitated by the oxidizing effect of the etching solution, which preferentially corroded the interface region between the rubber phase and the matrix, characterized by a lower crosslink density.

**Figure 11:**
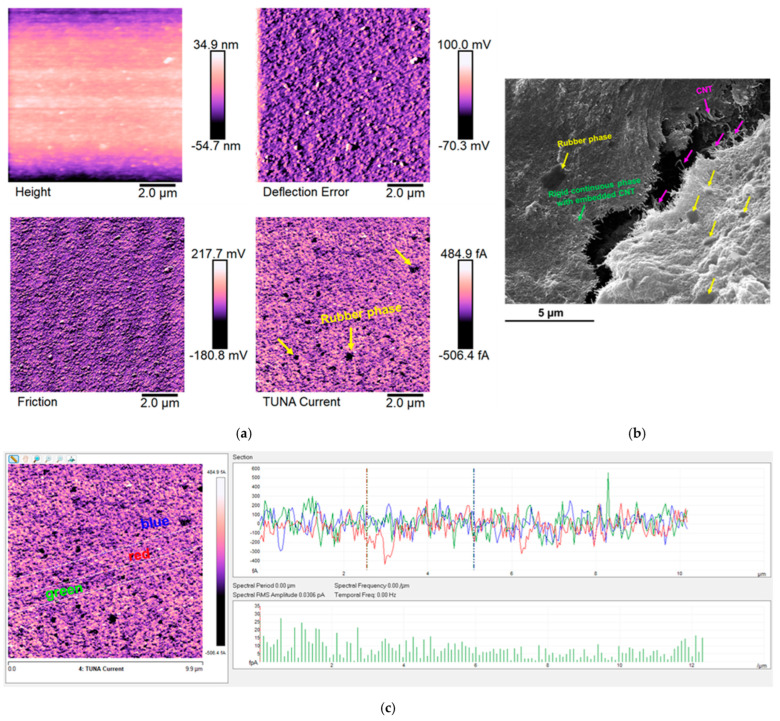
Morphological representation of epoxy sample Ep-CNT: (**a**) Height, Deflection Error, Friction, and TUNA Current images; (**b**) FESEM image; and (**c**) profile of the current variations.

**Figure 12:**
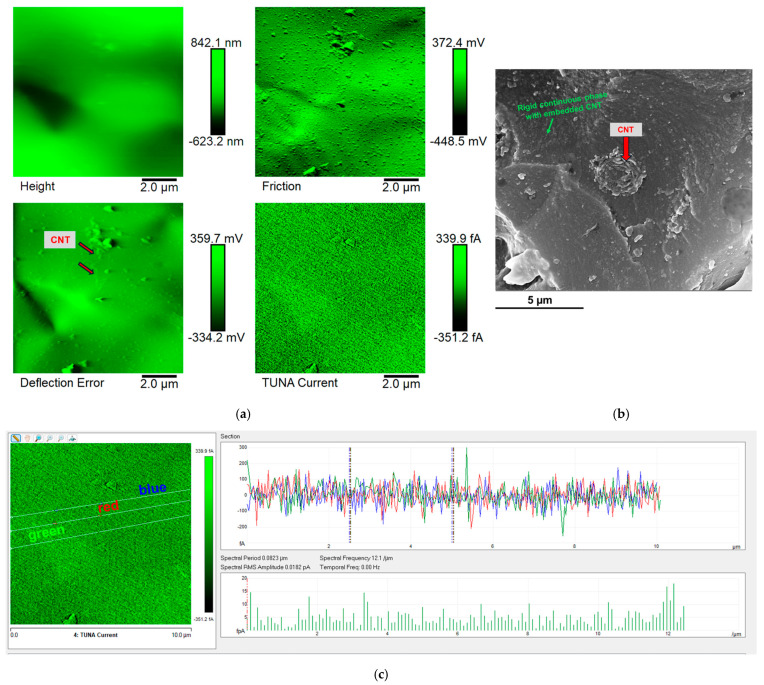
Morphological representation of epoxy sample Ep-CNT-D: (**a**) Height, Deflection Error, Friction, and TUNA Current images; (**b**) FESEM image; and (**c**) profile of the current variations.

**Figure 13:**
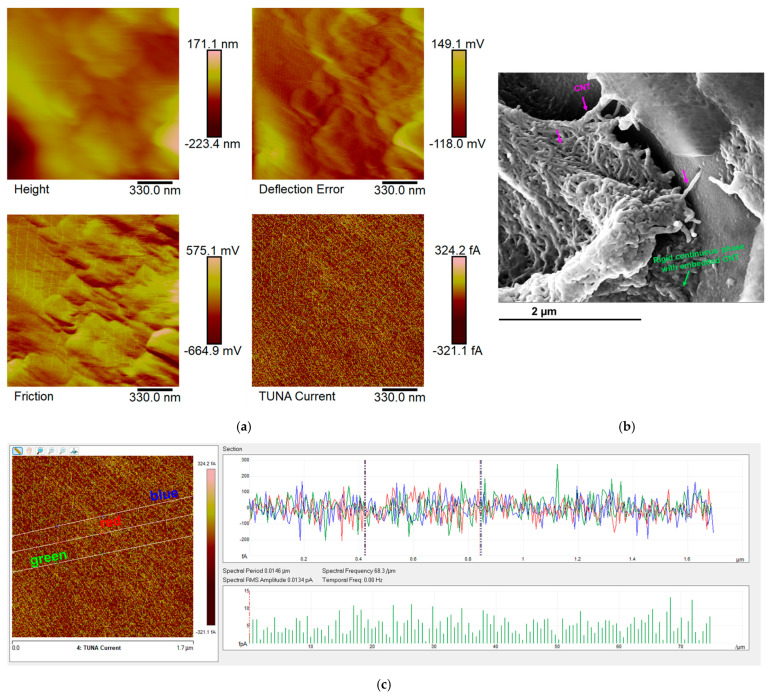
Morphological representation of epoxy sample Ep-CNT-T: (**a**) Height, Deflection Error, Friction, and TUNA Current images; (**b**) FESEM image; and (**c**) profile of the current variations.

**Figure 14:**
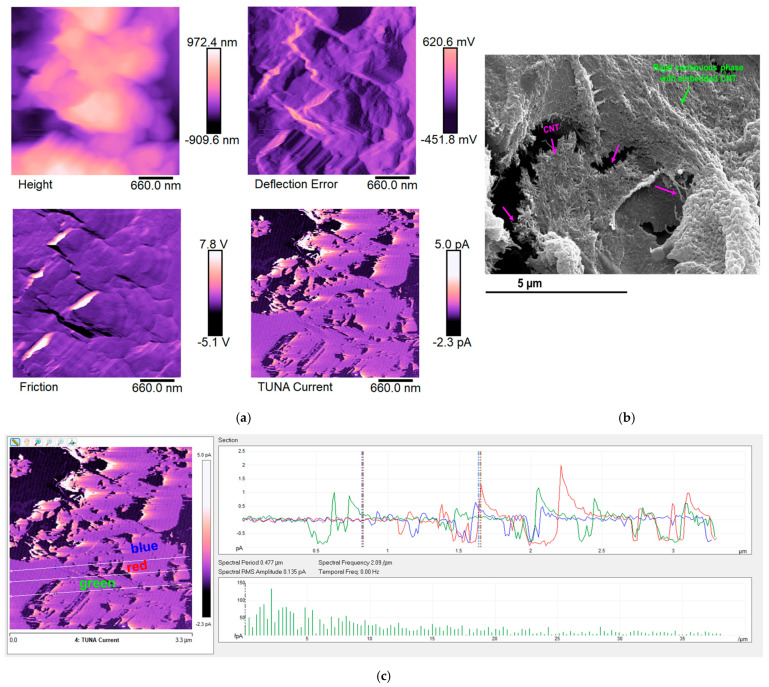
Morphological representation of epoxy sample Ep-CNT-M: (**a**) Height, Deflection Error, Friction, and TUNA Current images; (**b**) FESEM image; and (**c**) profile of the current variations.

The epoxy samples (Ep) loaded with carbon nanotubes (CNTs) alone and with both carbon nanotubes (CNTs) and self-healing fillers (D, T, and M) were morphologically characterized by FESEM and TUNA. In this regard, Figure 11, Figure 12, Figure 13 and Figure 14 show the morphological representation: (a) Height, Deflection Error, Friction, and TUNA Current images; (b) FESEM image; and (c) profile of the current variations in the Ep-CNT, Ep-CNT-D, Ep-CNT-T, and Ep-CNT-M, respectively. The simultaneous acquisition of multiple TUNA images, such as Height, Deflection Error, Friction, and TUNA Current, provides a comprehensive understanding of the sample’s morphological and electrical properties [66,67,68]. In fact, by acquiring these images simultaneously, we can correlate the morphological features (Height, Deflection Error, and Friction) with the electrical properties (TUNA Current) of the sample. This complementary information enables a more detailed and accurate characterization of the sample, facilitating the identification of specific morphological peculiarities and their impact on the sample’s electrical behavior. This approach turns out to be beneficial. More precisely, the Height image provides topographical information about the sample surface, helping to identify surface features, roughness, and the overall morphology of the sample. The Deflection Error image captures the deviations of the AFM cantilever from its setpoint during scanning, highlighting fine surface details and edges that may not be visible in the Height image alone. The Friction image measures the lateral forces experienced by the AFM tip as it scans the sample, providing insights into the sample’s surface friction properties that can be related to its material composition and surface interactions. The TUNA Current image maps the local electrical conductivity of the sample by measuring the tunneling current between the conductive AFM tip and the sample surface, revealing the distribution of conductive pathways and electrical heterogeneities within the sample.

Figure 11 presents TUNA and FESEM images of the toughened epoxy matrix Ep that has been infused with carbon nanotubes (CNTs), referred to as the Ep-CNT sample. These images enable the identification of the rubber phase, a rigid, continuous phase that contains embedded CNTs, creating percolated pathways. A nanofiller concentration of 0.5 wt% was selected for all analyzed samples, as it exceeds the electrical percolation threshold (EPT). Additionally, the carbon nanotubes are predominantly integrated within the resin. The carbon nanotubes are clearly well attached to the epoxy matrix, suggesting the existence of robust intermolecular interactions; however, they are not distributed within the rubber phase. The etching procedure has allowed us to highlight, in addition to an effective nanocharge dispersion, the good level of distribution of the carbon nanotubes throughout the resin, forming a solid, continuous, and conductive network that acts as a bridge between the various areas of the analyzed sample surface. The TUNA Current image of Figure 11a illustrates a gradient of colors on the scale bar, indicating areas of varying local electrical conductivity from the darkest to the lightest shades. For the sample Ep-CNT, which exhibited an electrical conductivity of 2.56 × 10^−2^ S/m [45], the electric current values were recorded between −506.4 fA and 484.9 fA. This demonstrates that the current flow, influenced by the tunnel effect of the conductive nanofiller, facilitates the effective transmission of electrical characteristics to the insulating matrix Ep, which was determined to have an electrical conductivity of 1.16 × 10^−14^ S/m [45].

Information on the dispersion of carbon nanotubes in the resin can also be obtained using the TUNA Current picture. From Figure 12, Figure 13 and Figure 14—which relate to the three epoxy samples loaded with carbon nanotubes and self-healing molecules, namely Ep-CNT-D, Ep-CNT-T, and Ep-CNT-M—we can observe a good distribution of the nanofiller, resulting in the formation of a continuous conductive network firmly anchored to the epoxy matrix through reversible non-covalent bonds. For the multifunctional self-healing systems Ep-CNT-D, Ep-CNT-T, and Ep-CNT-M, the electrical conductivity values of 1.15 × 10^–2^ S/m, 2.27 × 10^–4^ S/m, and 1.29 × 10^–2^ S/m were measured, respectively [45]. The obtained values of the electric current at the nanoscale are shown in the TUNA Current pictures of Figure 12a, Figure 13a, and Figure 14a. The electric current values for the sample Ep-CNT-D range from −351.2 fA to 339.9 fA (refer to Figure 12a). In the case of the sample Ep-CNT-T, the current values vary from −321.1 fA to 324.2 fA (see Figure 13a), while for the sample Ep-CNT-M, the current values span from −2.3 pA to 5.0 pA (illustrated in Figure 14a). Therefore, even in epoxy samples that incorporate self-healing fillers, the current flow induced by the tunnel effect, facilitated by the conductive nanofiller, significantly enhances the transmission of electrical characteristics to the insulating matrix.

The findings of surveying the local surface features through the use of the tunneling effect have demonstrated that the presence of self-healing fillers in the epoxy matrix containing carbon nanotubes does not result in a drop in the electric current values. The addition of self-healing fillers does not affect the conductive paths in the nanocomposites, and the good electric current values at the nanoscale level, along with the encouraging outcomes of the self-healing efficiency tests, validate the success of this strategic approach, which aims to create a multipurpose load-bearing material with the capacity for auto-repair. This strategic approach seems to be a valid premise for incorporating self-reactive functions based on the material’s electrical performance, such as self-sensing, de-icing, and/or anti-icing based on the Joule effect of the current passing through the composite material [69,70,71,72,73].

It is worth noting that the profile of the current fluctuations (green, red, and blue on the right) linked with the TUNA Current image (three white lines on the left) of Ep-CNT, Ep-CNT-D, Ep-CNT-T, and Ep-CNT-M is depicted in Figure 11c, Figure 12c, Figure 13c, and Figure 14c, respectively. By noting that the frequency of the current variations related to the filler/matrix interchanges along the three white lines is relatively constant for the three nanocomposites, it is easy to assess how evenly dispersed the nanocharged materials are within the matrix.

**Figure 15:**
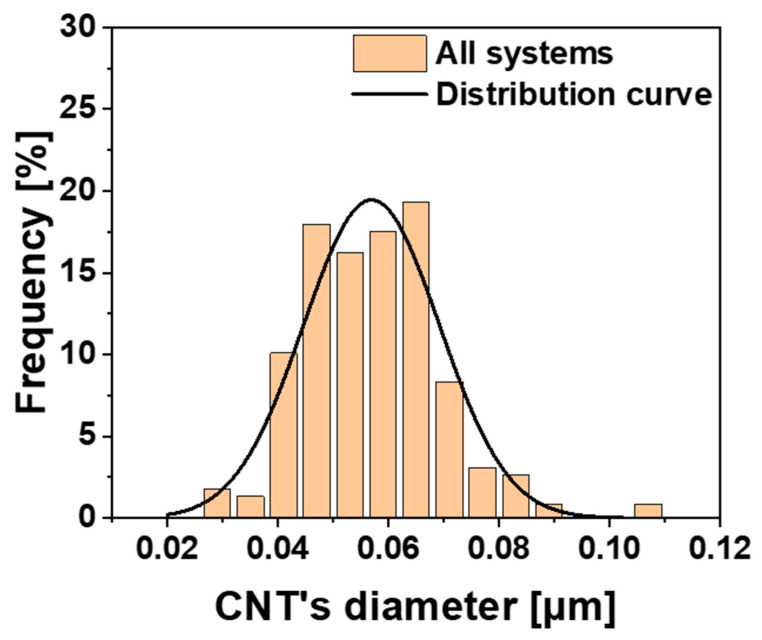
CNT size distribution. Histogram of relative frequency [%] versus CNT’s diameter [µm] obtained from all FESEM images of analyzed nanofilled systems Ep-CNT, Ep-CNT-D, Ep-CNT-T, and Ep-CNT-M, shown in Figure 11, Figure 12, Figure 13, and Figure 14, respectively.

Figure 15 shows the CNT size distribution. The histogram of the relative frequency [%] versus the CNTs’ diameter [µm] was obtained from all FESEM images of the analyzed nanofilled systems, Ep-CNT, Ep-CNT-D, Ep-CNT-T, and Ep-CNT-M, shown in Figure 11, Figure 12, Figure 13, and Figure 14, respectively.

Combining unfunctionalized carbon nanotubes (CNTs) with self-healing fillers represents a novel approach in developing multifunctional composites, offering distinct advantages over traditional functionalized CNT systems. This strategy addresses key challenges, such as maintaining electrical conductivity through the preservation of intrinsic properties, enhancing the healing efficiency, and achieving a uniform dispersion, thus leading to a significant advancement in the field of smart materials, as highlighted by recent studies [7,74,75,76,77,78,79]. These developments position the use of unfunctionalized CNTs as a promising alternative, addressing challenges like scalability and cost-effectiveness while maintaining a high performance. This study is very promising and is placed within a scenario of interesting technological implications in the research field, where the demand for increasingly high-performance materials is continuously evolving, and a significant challenge remains in developing key materials for multifunctional advanced systems suitably designed for real-world applications [80]. 

### 3.4. Self-Repairing Efficiency Evaluation Tests

**Figure 16:**
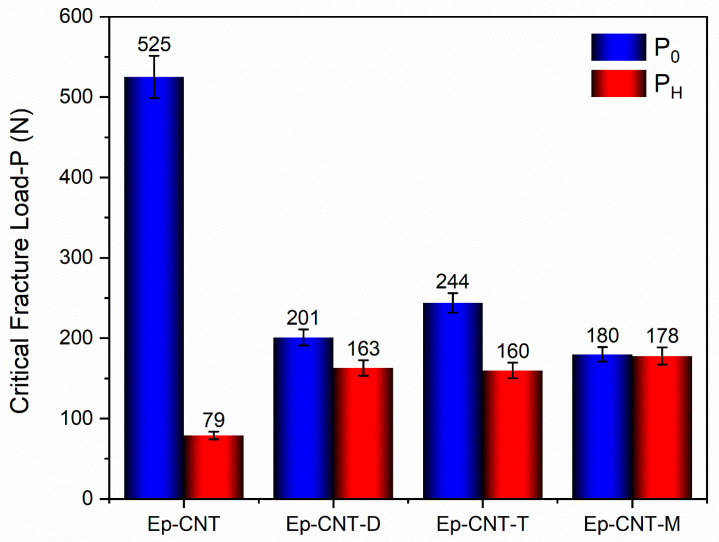
A histogram of the values of both the critical fracture load of the virgin samples ($P_{0}$) and the healed samples ($P_{H}$) for the cured nanocomposites: Ep-CNT, Ep-CNT-D, Ep-CNT-T, and Ep-CNT-M.

To evaluate the self-repairing functionality of the investigated nanocomposites, values of the critical fracture load of the virgin sample ($P_{0}$) and the healed samples ($P_{H})$ are reported in the histogram of Figure 16. Self-repairing efficiency evaluation tests were conducted for the nanocomposites loaded with self-healing fillers (Ep-CNT-D, Ep-CNT-T, and Ep-CNT-M). More precisely, the self-repairing efficiencies ($S_{H}$) were calculated using Equation (1), as described in Section 2.2.4. The values of $S_{H}$ higher than 65% were obtained for all the samples. Given the high healing efficiency values, we can assume that the different components (CNT and self-healing filler) work in concert to create a supramolecular network that can trigger the self-healing mechanisms if they interact synergistically. In this regard, it is worth noting that the epoxy matrix inherently possesses a modest ability to auto-repair due to the large number of -OH groups formed during the crosslinking process, which gives the structure its hardened form. The polymerized epoxy resins (cured with primary aromatic amines, like the DDS) can likewise experience reversible hydrogen interactions from the -OH groups. In contrast, for the reference sample Ep-CNT, the presence of CNTs alone results in a 15% increase in healing efficiency when self-healing fillers are not used [45]. Therefore, the simultaneous presence of carbon nanotubes (CNTs) and self-healing fillers (D, T, and M) determined an increase in the self-healing capacity of the nanocomposites. In fact, high $S_{H}$ values, i.e., 81.1% for Ep-CNT-D, 65.5% for Ep-CNT-T, and 98.9% for Ep-CNT-M, were recorded. The self-healing efficiency values of the nanocomposites are strictly connected to the chemical structure of the self-healing fillers, that is, to the type and quantity of functional groups present in the molecule that are able to participate in the hydrogen bonds. The highest $S_{H}$ value was exhibited by the sample loaded with the filler M, having more hydrogen bonding donor and acceptor sites than the other two compounds, D and T.

## 4. Conclusions

In this paper, we characterized the multifunctional self-healing supramolecular systems from both morphological and spectroscopic perspectives, using the TUNA technique and FT-IR, respectively.

The FT-IR analysis demonstrated the establishment of H-bonding interactions between the epoxy matrix and the self-healing fillers. A quantitative assessment of the hydrogen bonding interactions existing within the composites was made possible by the ratio R, which is defined as the area of the bonded O-H signal (A_OH-bond_) divided by the area of the free O-H signal (A_OH-free_). An elevated value of this ratio signifies a greater quantity of OH groups involved in hydrogen bonding interactions with self-healing fillers. The examined self-healing systems include unfunctionalized CNT nanoparticles, which can maintain their electrical properties more effectively, and compounds or molecules possessing groups within their structure that can facilitate the formation of hydrogen bonds. The compatibility of self-healing molecules with the hosting matrix is also effectively demonstrated through the TUNA morphological analysis which allows us to detect how the intrinsic ability to easily create the cumulative effects of the reversible interactions, based on hydrogen bonding in the matrix or epoxy network, translates into the formation of a continuous conductive network over the entire surface. From the TUNA analysis, we can observe a good distribution of nanofiller in all three samples (Ep-CNT-D, Ep-CNT-T, and Ep-CNT-M), resulting in the formation of a continuous conductive network strongly anchored to the epoxy matrix through reversible non-covalent bonds. From TUNA Current pictures, the electric current values at the nanoscale for the sample Ep-CNT-D range from −351.2 fA to 339.9 fA. In the case of the sample Ep-CNT-T, the current values vary from −321.1 fA to 324.2 fA, while for the sample Ep-CNT-M, the current values span from −2.3 pA to 5.0 pA. Therefore, even in epoxy samples that contain self-healing fillers, the current flow generated by the tunnel effect, enabled by the conductive nanofiller, substantially improves the transmission of electrical characteristics to the insulating matrix. The results of the electrical performance, which characterizes the conductive nanodomains of nanocharged resins, have shown that the incorporation of self-healing fillers in the epoxy matrix containing carbon nanotubes does not lead to a decrease in the electric current values. TUNA measurements validate the formation of a percolation pathway in the nanocomposites exceeding the electrical percolation threshold (EPT).

## Figures and Tables

**Table 1:** Information about the single components.

Compound	Description	References for Chemical Formula	Acronym
Tetra glycidyl methylene dianiline *	Tetrafunctional epoxy precursor, viscous liquid	[44]	E
1,4-butanedioldiglycidylether *	Reactive diluent, liquid	[44]	B
Carboxyl terminated butadiene acrylonitrile **	Toughening Elastomer, viscous liquid	[9,23]	C
4,4′-diaminodiphenyl sulfone *	Hardening agent, powder	[44]	H
Multiwall carbon nanotubes ***	Conductive filler (3100 Grade), powder	[9]	CNT
1,3-Dimethylbarbituric acid *	Self-healing filler, powder	[23,45]	D
2-Thiohydantoin *	Self-healing filler, powder	[23,45]	T
Murexide *	Self-healing filler, powder	[23,45]	M

* Purchased by Merck (Merck KGaA Darmstadt, Germany). ** Supplied by Hycar-Reactive Liquid Polymers (Huntsman Corporation, 10003 Woodloch Forest Dr, The Woodlands, TX 77380, USA). *** Purchased by Nanocyl S.A. (Sambreville, Belgium).

**Table 2:** Information about the samples analyzed.

Sample Composition	Description	Acronym
E + B	Uncured viscous liquid sample	EB
E + B + C	Uncured viscous liquid sample	EBC
E + B + self-healing filler	Uncured viscous liquid sample	EB-D
		EB-T
		EB-M
E + B + C + self-healing filler	Uncured viscous liquid sample	EBC-D
		EBC-T
		EBC-M
E + B + C + H	Oven-cured epoxy sample	Ep
E + B + C + H + CNT	Oven-cured epoxy sample	Ep-CNT
E + B + C + H + self-healing filler	Oven-cured epoxy sample	Ep-D
		Ep-T
		Ep-M
E + diluent + C + H + CNT + self-healing filler	Oven-cured epoxy sample	Ep-CNT-D
		Ep-CNT-T
		Ep-CNT-M

Oven-cured: two-stage curing cycle, namely 1 h at 125 °C and 3 h at 200 °C.

**Table 3:** Chemical formulas of self-healing fillers.

Self-Healing Filler	Structural Formula
D	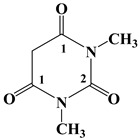
T	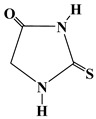
M	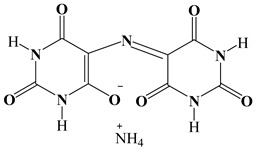

## Data Availability

Data are contained within the article.
